# Cerebral Small Vessel Disease in Elderly Patients With Vestibular Neuritis

**DOI:** 10.3389/fneur.2022.818533

**Published:** 2022-03-31

**Authors:** Fieke K. Oussoren, Louise N. F. Poulsen, Joost J. Kardux, Tjard R. Schermer, Tjasse D. Bruintjes, Roeland B. van Leeuwen

**Affiliations:** ^1^Apeldoorn Dizziness Centre, Gelre Hospital, Apeldoorn, Netherlands; ^2^Department of Otorhinolaryngology, Leiden University Medical Center, Leiden, Netherlands; ^3^Department of Radiology, Gelre Hospital, Apeldoorn, Netherlands

**Keywords:** stroke, vestibular neuritis (VN), MRI, white matter hyper intensities, vascular etiology

## Abstract

**Background:**

Acute audiovestibular loss is a neurotologic emergency of which the etiology is frequently unknown. In vestibular neuritis a viral genesis is expected, although there is insufficient evidence to support viruses as the only possible etiological factor. In sudden deafness, a vascular etiology has been proposed in elderly patients, since cardiovascular risk factors are more frequently present and a higher risk of developing a stroke was seen compared to the general population. So far, very little research has been carried out on vascular involvement in elderly patients with vestibular neuritis. Cardiovascular risk factors have a positive correlation with cerebral small vessel disease, visible as white matter hyperintensities, brain infarctions, microbleeds and lacunes on MRI. The presence of these characteristics indicate a higher risk of developing a stroke.

**Aim:**

We investigated whether elderly patients with vestibular neuritis have a higher prevalence of vascular lesions on MRI compared to a control cohort.

**Materials and Methods:**

Patients of 50-years and older, diagnosed with vestibular neuritis in a multidisciplinary tertiary referral hospital, were retrospectively reviewed and compared to a control cohort. The primary outcome was the difference in cerebral small vessel disease on MRI imaging, which was assessed by the number of white matter hyperintensities using the ordinal Fazekas scale. Secondary outcomes were the presence of brain infarctions on MRI and the difference in cardiovascular risk factors.

**Results:**

Patients with vestibular neuritis (*N* = 101) had a 1.60 higher odds of receiving a higher Fazekas score than the control cohort (*N* = 203) (*p* = 0.048), there was no difference in presence of brain infarctions (*p* = 1.0). Hyperlipidemia and atrial fibrillation were more common in patients experiencing vestibular neuritis.

**Conclusion:**

We found a positive correlation of white matter hyperintensities and VN which supports the hypothesis of vascular involvement in the pathophysiology of vestibular neuritis in elderly patients. Further prospective research is necessary to confirm this correlation.

## Introduction

Acute audiovestibular loss is a neurotologic emergency. The cause of vestibular neuritis (VN), sudden sensorineural hearing loss (SSNHL) or labyrinthitis is unknown in a significant number of cases (1, 2).

Recently, research has focused on a possible vascular cause of audio and vestibular loss (3–6). An acute onset of symptoms and frequently unilateral presentation resemble acute cardiovascular diseases.

Very little information is available on vascular involvement in VN. It is believed to be an inflammatory disorder of viral origin (7). However, patients do not show clinical benefit from antiviral therapy or corticosteroid use (8, 9). The question is whether VN might have a different etiology and, consequently, a different therapy should be applied.

Because of its relatively high incidence, SSNHL has been studied frequently. Compared to the general population, cardiovascular risk factors are more frequently present in patients with SSNHL (3, 10, 11). Subsequently, several authors have investigated the chance of developing a stroke in patients with SSNHL. These studies show that, after correction for age and other cardiovascular risk factors, patients with sudden hearing loss have a 1.26–2.02 higher chance of developing a stroke than the general population (12–15).

A vascular compromised brain raises the chance of developing a stroke. On magnetic resonance imaging (MRI) imaging, cerebral small vessel disease (CSVD), visible as white matter hyperintensities (silent), brain infarctions and microbleeds have shown to be indicative of developing stroke (16–19).

With this study we investigated whether patients with VN have more CSVD on MRI compared suggesting a vascular involvement in the pathophysiology of VN.

## Materials and Methods

### Setting

This retrospective case-control study was based upon hospital records from patients either visiting the emergency departments of Gelre Hospital Apeldoorn and Zutphen, the outpatient neurology clinic or the Apeldoorns Dizziness Centre (ADC), located in Gelre hospital. The ADC serves as a tertiary referral center that specializes in the diagnostic and therapeutic workup of dizziness. It is a multidisciplinary center involving the Neurology, Clinical neurophysiology and Otorhinolaryngology departments of the Gelre Hospital Apeldoorn. This retrospective cohort study gained ethical approval from the local ethics committee at Gelre Hospital Apeldoorn.

### Cohorts

A study cohort was compiled of patients diagnosed with vestibular neuritis between January 2010 and March 2021 who received an MRI cerebrum. Patients either presented at the emergency departments of both Gelre hospitals or at the outpatient Apeldoorn dizziness centre.

Vestibular neuritis was defined as a single episode of acute, severe vertigo lasting for at least 24 h in the absence of auditory symptoms or neurological symptoms, with or without loss of vestibular function measured with caloric testing. VN was distinguished from acute stroke either by a positive head impulse test, by the presence of unidirectional horizontal nystagmus, by the absence of skew deviation (HINTS) or by the absence of an infarction on MRI. At the emergency departments patients the diagnostic tests were performed by different physicians.

The control cohort was compiled of patients who either visited the outpatient neurological department with facial pain, suspected for trigeminal neuralgia, or patients who visited the ADC with recurrent episodes of spontaneous vertigo lasting several seconds, suggestive of vestibular paroxysmia. All patients received an MRI cerebrum to rule out the presence of an intracranial neoplasm.

Exclusion criteria were age 49 years or younger and a history of cerebrovascular accident or transient ischemic attack. If during follow-up the type of dizziness changed and did not meet the aforementioned criteria of VN, these patients were excluded. Patients were also excluded if the MRI was performed more than a year after presentation at either the emergency department or the outpatient dizziness clinic.

### MRI Protocol

An MRI was suitable for radiological assessment of white matter hyperintensities and brain infarctions if at least one sequence of the entire brain, either FLAIR or T2, was available. The imaging was performed using a 1.5 Tesla MRI scanner. The cerebral sequence was depicted with a slice thickness of 5 mm. Twenty-three MRI scans were performed elsewhere and uploaded in the Gelre Hospital database, two of these scans had a slice thickness of 4 mm, and the remaining had a slice thickness of 5 mm. The type of MRI scanner used for these external MRIs could not be retrieved.

### Outcomes

**Figure 1:**
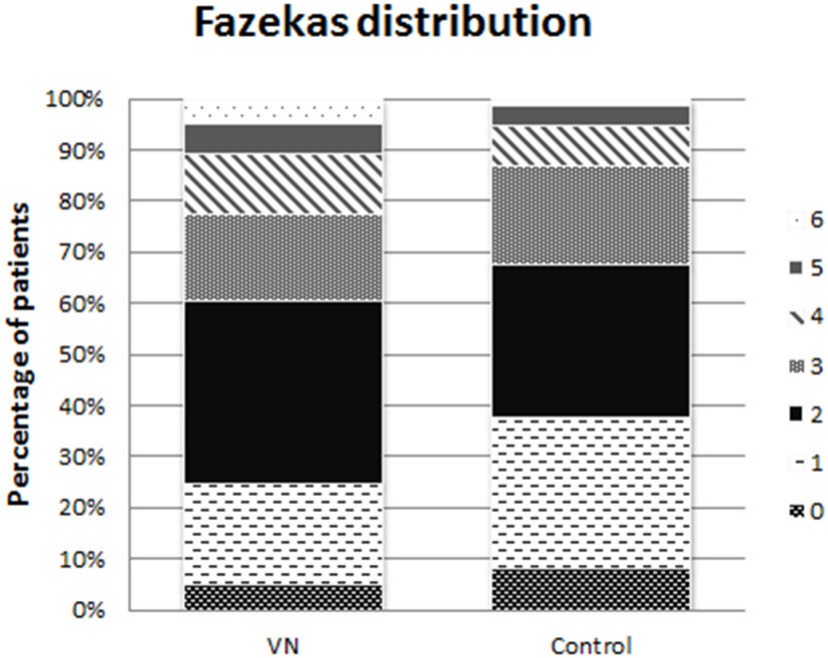
Fazekas scale for MRI imaging. The figure displays hyperintensities in the deep white matter (upper row) and periventricular (lower row).

The primary outcome was the degree of cerebral vascular damage assessed on MRI imaging by measuring the Fazekas score. The Fazekas score is a validated diagnostic tool for assessing the severity of white matter hyper-intensities both periventricular and in the deep white matter with a possible score from 0 to 6, where 0 means no hyperintensities present, (see Figure 1).

A secondary outcome was the presence of brain infarctions on MRI imaging. Brain infarctions were defined by lesions of the brain of at least 3 mm with a cerebrospinal fluid appearance on all MRI sequences, differentiable from leukariosis and dilated Virchow-Robinson spaces that did not result in prior neurological deficits (20).

Another secondary outcome was the difference in the presence of the cardiovascular risk factors; smoking, hypertension, hyperlipidemia, diabetes, a history of myocardial infarction and atrial fibrillation between the SSNHL and control cohort. The following assumptions were made in the identification of cardiovascular risk factors. Hypertension and diabetes were defined as being present either by having a positive medical history or if medication for these conditions were used. In case no complete medical history had been obtained, the variable was defined as missing. Hyperlipidemia was defined as being present when the patient had a positive medical history of dyslipidemia, used statins, or had an elevated total cholesterol level of >4.9 mmol/L within a month before or after presentation at our dizziness centre.

### Assessment

MRI imaging was assessed by two radiologists separately, LP and JK. The two radiologists involved in this study have multiple years of experience in examining MRI imaging of the head and neck. To limit observer bias, both radiologists were blinded for the patients' characteristics or study arm.

If there was a difference in Fazekas rating between the two raters, the following rules were applied. If the difference was 1 rank, the highest rank was then applied. If the difference was 2 ranks or more, the radiologists reviewed the case together until consensus was reached.

Baseline characteristics, data from diagnostic tests and MRI ratings were gathered and de-identified in a Castor electronic database (CastorECD, Amsterdam, The Netherlands).

### Rater Reliability Testing

Inter- and intra-rater reliability for the two radiologists was assessed for the Fazekas rating scale using the present control cohort plus a cohort of patients who suffered from sudden sensorineural hearing loss. All patients were rated by the same raters involved in the present study. Sample size calculation showed that thirty patients had to be rated twice by both raters to evaluate the intra-rater reliability. A weighted Cohens' kappa coefficient was calculated using linear weighting, where the difference between low and high ratings is of equal importance.

### Statistical Analysis

Continuous variables were described using the following summary descriptive statistics: number of non-missing values, mean and standard deviation in case of normally distributed data or median and interquartile ranges in case of non-normally distributed data.

Categorical variables were described using frequencies and percentages. Percentages were calculated on the number of non-missing observations.

Ninety-five confidence intervals were calculated when applicable. Statistical testing was performed two-sided at a 0.05 significance level.

Differences in the ordinal ranking of the Fazekas scale between the two cohorts were calculated using the Mann-Whitney U test for ordinal non-paired data.

Because the presence of brain infarctions is a binary variable, the difference between the cohorts was compared using the Chi-square test.

Ordinal logistic regression analysis was performed to compare the outcomes between the cohorts while adjusting for the potential confounders: age, hypertension, hyperlipidemia, diabetes, a medical history of MI, smoking, gender, an outpatient or inpatient presentation, the presence of vestibular loss with an abnormal video head impulse testing or abnormal caloric testing and the type of MRI sequence used.

## Results

### Patient Characteristics

A total of 101 patients with VN were included. The control cohort consisted of 203 patients, 149 suspected cases of trigeminal neuralgia and 54 suspected cases of vestibular paroxysmia. In the vestibular neuritis cohort, 50 patients were diagnosed upon presentation at the emergency department while 51 patients were diagnosed at the outpatient clinic of the Apeldoorn Dizziness Centre. None of the VN patients demonstrated bilateral vestibular dysfunction.

Baseline characteristics of both cohorts are displayed in Table 1. The mean age did not differ significantly between both cohorts. Both study cohorts consisted of more women than men.

**Table 1:** Patient characteristics.

	**VN (*N* = 101)**	**Control (*N* = 203)**	**Missing**	***P*-value**
Age [mean, (SD)]	64 (9.8)	63 (9.4)	0	0.423
^*^50–60	42 (41.6)	100 (49.3)	0	0.469
^*^61–70	28 (27.7)	56 (27.6)	0	
^*^71–80	28 (27.7)	41 (20.2)	0	
^*^>80	3 (3.0)	6 (3.0)	0	
Gender			0	0.460
Male	56 (55.4)	123 (60.6)	
Female	45 (44.6)	80 (39.4)	
Prior myocardial infarction	4 (4.0)	7 (3.4)	0	1.000
Anticoagulant use	12 (11.9)	21 (10.3)	0	0.700
Smoking			27	0.073
Former	21 (22.6)	25 (13.6)	
Yes	6 (6.5)	23 (12.5)	
Hypertension	35 (36)	67 (33.3)	6	0.700
Hyperlipidemia	52 (51.5.0)	63 (31.0)	0	0.001
Diabetes	7 (6.9)	20 (9.9)	0	0.522
Atrial fibrillation	8 (7.9)	4 (2.0)	0	0.023

*Patient characteristics of 101 patients with vestibular neuritis and a control cohort of 203 patients displayed in numbers and percentages. For age the mean and standard deviation are displayed. N, number; SD, standard deviation; VN, vestibular neuritis*.

* *Age stratified by decades*.

In patients with VN, hyperlipidemia and atrial fibrillation were significantly more common. Also a medical history of myocardial infarction, smoking and hypertension were more frequently present in the VN cohort, though not statistically significant. Diabetes was more frequently present in the control cohort, also not statistically significant.

In case of presentation at the emergency department, the MRI was made after 24 h in only six cases. All other patients received an MRI within 24 h up to several days after the onset of symptoms. The MRI of 31 (30.7%) patients in the VN cohort was assessed using a T2 sequence compared to 106 (52.2%) in the control cohort (*p* < 0.001).

### Rater-Reliability Testing

All 328 MRI scans were reviewed by both raters. In 201 cases both radiologists gave the same Fazekas score, in 103 cases they differed one point and in 24 cases the difference in score given was two points or more. This resulted in a kappa-coefficient of 0.74 for inter-rater reliability.

Thirty subjects were rated twice by each rater, which resulted in a weighted kappa-coefficient of 0.80 and 0.82 for rater 1 and 2, respectively, suggesting a near-perfect agreement for each rater.

### Fazekas Score

**Figure 2:**
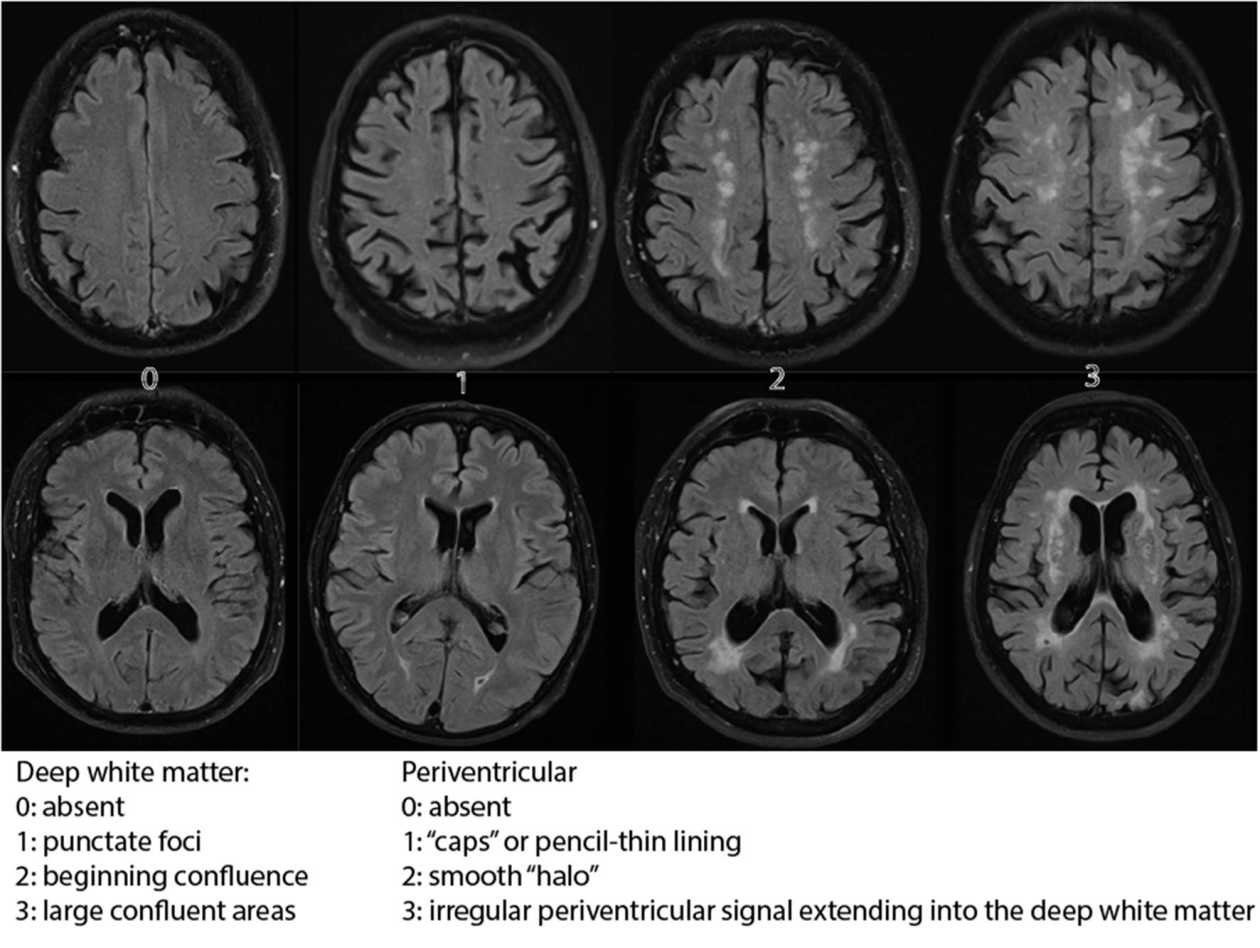
Fazekas distribution. The distribution of the Fazekas scale score 1 up to 6 over the both cohorts displayed in percentages. VN, vestibular neuritis.

The distribution of Fazekas ratings in both cohorts is displayed in Figure 2.

The modus of the Fazekas score was 2 in both cohorts. The group that received Fazekas 5 and 6 form a larger proportion of cases in the VN cohort than in the control cohort. Therefore, the ordinal differed statistically significant (*p* = 0.023).

### Ordinal Regression Analysis

Table 2 displays the results of the ordinal regression analysis. In a univariate regression analysis only the variables neuritis, hypertension and age all significantly increased the risk of a higher Fazekas score. The remaining cardiovascular risk factors and diagnostic characteristics did not influence the Fazekas score.

**Table 2:** Ordinal regression analysis.

**Variable**	**Univariate**			**Multivariate**		
	**Odds**	**Sig**	**95% CI**	**Odds**	**Sig**	**95% CI**
Neuritis	2.10	0.012^*^	1.26–3.83	1.6	0.048^*^	1.01–2.42
Age	1.11	0.000^*^	1.08–1.13	1.10	0.000^*^	1.07–1.13
Diabetes	0.96	0.909	0.47–1.94			
Gender	1.11	0.628	0.74–1.66			
History of MI	2.46	0.100	0.84–7.15			
Hyperlipidemia	1.22	0.358	0.81–1.85			
Hypertension	2.48	0.000^*^	1.60–3.84	1.52	0.067	0.97–2.39
Smoking	1.00	0.997	0.73–1.37			
Abnormal caloric testing	1.02	0.928	0.63–1.66			
Abnormal video-HIT	1.17	0.717	0.50–2.73			
Outpatient presentation	0.85	0.648	0.42–1.71			
MRI Sequence (FLAIR)	1.48	0.058	0.99–2.22			

*Regression analysis for Fazekas score. FLAIR, Fluid Attenuated Inversion Recovery; HIT, head impulse test; CI, Confidence interval; MI, myocardial infarction; MRI, Magnetic Resonance Imaging; Sig, significance*;

* *Significant at level p < 0.05*.

After adding hypertension and age to the model, patients with vestibular neuritis had a 1.60 (95%CI 1.01–2.42, *p* = 0.048) higher odds of having a higher Fazekas score. Age also significantly increased the odds of white matter hyperintensities on MRI.

### Brain Infarctions

Brain infarctions were present in 10 (9.9%) cases with VN and 21 (10.4%) cases in the control cohort. The difference between the two cohorts was not significant (*p*-value = 1.0).

In both cohorts, most brain infarctions were located in the deep white matter, in five patients from the VN cohort and seven from the control cohort. The cerebellum was affected in three patients with VN and seven controls. In five control patients an infarction was located in the basal ganglia, while none of the patients with VN had lesions in this region. In only one patient with vestibular neuritis a cortical infarction was seen, compared to four patients in the study cohort. In both cohorts the brainstem was not affected. Due to the limited number of cerebral infarctions, a regression analysis was not performed.

## Discussion

We found a positive correlation between VN and CSVD on MRI imaging. Patients with VN had a 1.60 odds of receiving a higher Fazekas rating compared to the control cohort. Very few brain infarctions were seen in both groups resulting in no significant difference between the study cohorts. All cardiovascular risk factors, apart from diabetes, were more frequently present in the study cohort. However, the difference in prevalence was only statistically significant for hyperlipidemia and atrial fibrillation.

Literature on vascular involvement in the pathophysiology of VN is limited. We based our hypothesis upon the following train of thought. VN is generally expected to be the result of inflammation secondary to viral infection. VN often has a viral prodrome and latent herpes simplex virus type 1 has been detected in human vestibular ganglia with PCR (21). Also, postmortem studies showed atrophy of the vestibular nerve and sensory epithelium, similar to pathological alterations seen in inner ear infections with measles (22–25). Nevertheless, corticosteroid and antiviral therapy have failed to show clinical benefit in the treatment of VN (8, 9).

The current treatment in the Netherlands is symptomatic with vestibular suppressants and anti-emetics. The question is whether VN can be solely contributed to a viral infection or if a different pathophysiology is probable. Since the ear and vestibular organ have limited collateral blood supply, it is particularly vulnerable to blood pressure dysregulation or acute occlusion.

Blood pressure dysregulation (BPD) in the vestibulocochlear blood circulation was first described by Fisch et al. (26). Cochlear atherosclerosis, related to cardiovascular risk factors as age, hypertension, diabetes, hyperlipidemia and smoking, results in a pathologic alteration in the composition of the arteries and arterioles. Hyalinosis causes thickening of the tunica adventitia, while the number of fibromuscular cells is reduced, which induces a decrease in adrenergic regulation (27). Meanwhile, due to arteriosclerosis, the internal caliber of the arteriole is reduced. This combination of blood pressure dysregulation secondary to reduced adrenergic regulation and shrinking of the arterial lumen results in damage to the vestibular nerve fibers (28, 29).

Acute onset vertigo or sudden sensorineural hearing loss, with or without vertigo, can also be caused by acute occlusion somewhere along the course of the anterior inferior cerebellar artery. After studying the vascular structure of the inner ear, Tange et al. came up with a theoretical flowchart depicting obstruction lines and the expected symptoms caused by this obstruction (30). In case of acute arterial occlusion, one would expect a patient to present with combined audiovestibulopathy, because of the common vascular supply to the cochlea and vestibule by the internal auditory artery.

A third argument supporting a vascular hypothesis of VN is based upon microvascular occlusion secondary to inflammation either due to viral infection or auto-immune response. Freedman et al. found a significantly elevated expression of CD40 positive monocytes and macrophages in patients with VN (31). These cells are known to cause platelet-monocytes aggregates that might cause thrombotic changes in the vascular system. A significantly increased expression of cyclooxygenase−2 (COX-2) was also found in patients with VN (32). This enzyme is responsible for vasodilatation and is generally present in the peripheral blood mononuclear cells (PBMC's) of patients with cardiovascular comorbidity (32, 33). It is suggested that the proinflammatory activation of PBMC's and elevation of CD 40 expression reduces the microvascular perfusion of the vestibular organ by increased thrombotic events, resulting in loss of function of the vestibular organ.

Since the cochlea is supplied by the same vascular system as the vestibular organ, a similar hypothesis of vascular compromise in the origin SSNHL has gained considerable attention. Hypoperfusion due to arteriosclerosis and BPD is thought to result in damage of the stria vascularis and subsequent hearing loss. Ciorba et al. and Fusconi et al. have investigated the presence of white matter hyperintensities as an indicator of cerebral small vessel disease in patients with SSNHL (34, 35). Ciorba et al. found no difference in the incidence of WMH, they did, however, find a correlation between more WMH and a poorer hearing recovery (34). Fusconi et al. found more WMH in individuals aged 40–60 in the SSNHL subset and also correlated this to a poorer rearing recovery (35). Several other authors found a higher incidence of stroke following SSNHL than in the general population (12, 14, 15).

Oron et al. investigated the presence of cardiovascular risk factors in patients with VN compared to the general population (36). They found a significantly higher presence of dyslipidemia, hypertension, diabetes, ischemic heart disease, prior CVA/TIA, cigarettes smoking, and obesity when compared to healthy controls. Their study cohort consisted of 160 patients with VN with a mean age of 56 years old, which is a larger but also younger study populations than our cohort. Age was a significant contributor of the prevalence in cardiovascular risk factors. For this exact reason our study population consisted only of patients 50 years of age or older. Since age is known to be an important risk factor for both CSVD and the risk of developing stroke, we corrected for age in a multivariate regression analysis. After correction, patients in the VN cohort still had increased odds of having a higher degree of white matter hyperintensities.

Chung et al. also investigated a possible vascular etiology in VN (37). They compared metabolic syndrome scores and arterial stiffness, using brachial ankle pulse wave velocity, between patients with VN and controls (37). They found an increased arterial stiffness and hypothesized that this increase might reflect endothelial dysfunction and microvascular compromise in patients with VN. Since arterial stiffness can affect small vessel in the brain it could lead to cerebral small vessel disease (38).

Adamec et al. previously demonstrated that white matter supratentorial lesions and older age reduced the odds of clinical recovery after VN (39). They speculated that because of interaction with central compensatory mechanisms, white matter lesions can influence the clinical recovery after VN (39).

This is the first study to compare cerebral small vessel disease in elderly patients with VN to a control cohort. The positive association that was found could have significant clinical implications since cerebral vascular damage increases the risk of developing cardiovascular disease. According to Fazekas et al. cerebral microbleeds, white matter hyperintensities, silent brain infarctions and lacunes are indicators of cognitive impairment and stroke (16–18). The presence of these indicators in patients with VN should henceforth caution the physician for vascular involvement in VN.

We need to address some limitations that are unavoidable as a consequence of the retrospective study design. As explained in the method section, several assumptions were made in the recording of cardiovascular risk factors. This could have resulted in some underestimation of the presence of these risk factors. However, there is no reason to suspect that this underestimation differed between both cohorts.

Also, we did not use a standardized sequence schedule for MRI assessment. In some patients, the Fazekas score and presence of brain infarctions were evaluated on a FLAIR sequence, while in others a T2 sequence was used. White matter hyperintensities and brain infarctions can be seen on both sequences and the slice-thickness did not differ between patients. Furthermore, the type of MRI sequence used did not correlate with the Fazekas score in the regression analysis.

As opposed to Menière's disease or vestibular migraine, there are no universally accepted criteria for the clinical diagnosis of vestibular neuritis. HINTS was proven to be superior to MRI in differentiating VN from a stroke in the acute phase (40). The accuracy of diagnosing VN is determined predominantly by the physicians' experience in performing and interpreting these diagnostic tests. Since the patients presenting in the emergency departments were diagnosed by different physicians with different levels of competence in performing these diagnostic tests, this might have influenced the selected study cohort. In a univariate regression analysis, the outpatient presentation did not influence the degree of white matter hyper intensities in patients with vestibular neuritis.

Also, not all patients with VN receive an MRI. An MRI is usually performed to exclude a central cause of the dizziness. Patients who received an MRI might have had more severe dizziness than patients who did not receive imaging or had an unclear diagnosis at first. This might have resulted in some selection bias.

Finally, resilience, the capacity to cope with brain pathology is a factor that can be influenced by cerebral small vessel disease (28). Patients with a high degree of cerebral small vessel disease might develop more severe symptoms after vestibular neuritis than patients with limited small vessel disease, since they have limited brain reserve, i.e., white matter structural integrity, to compensate the loss of vestibular function. While high cognitive reserve, i.e., educational attainment and IQ, can attenuate the effect of cerebral small vessel disease on cognitive function (41). In this study the premorbid cognitive ability was not tested and could therefore be a confounder. Future prospective studies should implement a baseline cognitive function test.

Regardless of these limitations, patients with vestibular neuritis presented more often with CSVD than the control cohort, supporting the hypothesis of vascular involvement in the pathophysiology of VN in a subset of elderly patients. These results, however, cannot be extrapolated to younger patients.

The next step would be to confirm a positive correlation of VN with cardiovascular risk factors and CSVD in a prospective setting, without the limitations of a retrospective study design. The time course of vertigo should be determined in prospective work, since a sudden onset would favor a vascular hypothesis whereas acute onset with evolution to peak intensity over 1 up to 3 days would better support a post-infectious or inflammatory mechanism.

Further research should then focus on whether elderly patients with vestibular neuritis have a higher chance of developing cardiovascular disease and should receive cardiovascular risk management or anticoagulant therapy.

## Data Availability Statement

The original contributions presented in the study are included in the article/supplementary files, further inquiries can be directed to the corresponding author/s.

## Ethics Statement

The studies involving human participants were reviewed and approved by Local Ethics Committee Gelre Hospital Apeldoorn. Written informed consent for participation was not required for this study in accordance with the national legislation and the institutional requirements.

## Author Contributions

FO is responsible for the data collection, data analysis, and overall integrity of the paper. LP and JK are responsible for the radiological assessment and the integrity of the radiological part of the materials and method section. TS is responsible, together with FO, for the statistical analysis and the integrity of this section in the paper. TB and RL are responsible, together with FO, for the study design, writing the article, and overall integrity of the paper. All authors contributed to the article and approved the submitted version.

## Conflict of Interest

The authors declare that the research was conducted in the absence of any commercial or financial relationships that could be construed as a potential conflict of interest.

## Publisher's Note

All claims expressed in this article are solely those of the authors and do not necessarily represent those of their affiliated organizations, or those of the publisher, the editors and the reviewers. Any product that may be evaluated in this article, or claim that may be made by its manufacturer, is not guaranteed or endorsed by the publisher.
